# Association of Lipid-Related Genetic Variants with the Incidence of Atrial Fibrillation: The AFGen Consortium

**DOI:** 10.1371/journal.pone.0151932

**Published:** 2016-03-21

**Authors:** Faye L. Norby, Samuel Adamsson Eryd, Maartje N. Niemeijer, Lynda M. Rose, Albert V. Smith, Xiaoyan Yin, Sunil K. Agarwal, Dan E. Arking, Daniel L. Chasman, Lin Y. Chen, Mark Eijgelsheim, Gunnar Engström, Oscar H. Franco, Jan Heeringa, George Hindy, Albert Hofman, Pamela L. Lutsey, Jared W. Magnani, David D. McManus, Marju Orho-Melander, James S. Pankow, Gull Rukh, Christina-Alexandra Schulz, André G. Uitterlinden, Christine M. Albert, Emelia J. Benjamin, Vilmundur Gudnason, J. Gustav Smith, Bruno H. C. Stricker, Alvaro Alonso

**Affiliations:** 1 Division of Epidemiology and Community Health, School of Public Health, University of Minnesota, Minneapolis, Minnesota, United States of America; 2 Department of Clinical Sciences, Lund University, Malmö, Sweden; 3 Department of Epidemiology, Erasmus Medical Center—University Medical Center, Rotterdam, The Netherlands; 4 Division of Preventive Medicine, Department of Medicine, Brigham and Women's Hospital, Harvard Medical School, Boston, Massachusetts, United States of America; 5 Icelandic Heart Association, Research Institute, Kopavogur, Iceland; 6 The University of Iceland, Reykjavik, Iceland; 7 Cardiology and Preventive Medicine Sections, Department of Biostatistics, Boston University School of Medicine, Boston, Massachusetts, United States of America; 8 Icahn School of Medicine, Mount Sinai Heart Center, New York, New York, United States of America; 9 McKusick-Nathans Institute of Genetic Medicine, Johns Hopkins University School of Medicine, Baltimore, Maryland, United States of America; 10 Cardiac Arrhythmia Center, Cardiovascular Division, Department of Medicine, University of Minnesota Medical School, Minneapolis, Minnesota, United States of America; 11 Department of Internal Medicine, Erasmus Medical Center—University Medical Center, Rotterdam, The Netherlands; 12 Cardiology and Preventive Medicine Sections, Department of Medicine, Boston University School of Medicine, Boston, Massachusetts, United States of America; 13 The National Heart, Lung and Blood Institute’s and Boston University’s Framingham Heart Study, Framingham, Massachusetts, United States of America; 14 Departments of Medicine and Quantitative Health Sciences, University of Massachusetts Medical School, Worcester, Massachusetts, United States of America; 15 Division of Cardiovascular Medicine, Department of Medicine, Brigham and Women's Hospital, Harvard Medical School, Boston, Massachusetts, United States of America; 16 Department of Epidemiology, Boston University School of Public Health, Boston, Massachusetts, United States of America; 17 Department of Cardiology, Lund University, Lund, Sweden; 18 Inspectorate of Health Care, Utrecht, the Netherlands; University at Buffalo, UNITED STATES

## Abstract

**Background:**

Several studies have shown associations between blood lipid levels and the risk of atrial fibrillation (AF). To test the potential effect of blood lipids with AF risk, we assessed whether previously developed lipid gene scores, used as instrumental variables, are associated with the incidence of AF in 7 large cohorts.

**Methods:**

We analyzed 64,901 individuals of European ancestry without previous AF at baseline and with lipid gene scores. Lipid-specific gene scores, based on loci significantly associated with lipid levels, were calculated. Additionally, non-pleiotropic gene scores for high-density lipoprotein cholesterol (HDLc) and low-density lipoprotein cholesterol (LDLc) were calculated using SNPs that were only associated with the specific lipid fraction. Cox models were used to estimate the hazard ratio (HR) and 95% confidence intervals (CI) of AF per 1-standard deviation (SD) increase of each lipid gene score.

**Results:**

During a mean follow-up of 12.0 years, 5434 (8.4%) incident AF cases were identified. After meta-analysis, the HDLc, LDLc, total cholesterol, and triglyceride gene scores were not associated with incidence of AF. Multivariable-adjusted HR (95% CI) were 1.01 (0.98–1.03); 0.98 (0.96–1.01); 0.98 (0.95–1.02); 0.99 (0.97–1.02), respectively. Similarly, non-pleiotropic HDLc and LDLc gene scores showed no association with incident AF: HR (95% CI) = 1.00 (0.97–1.03); 1.01 (0.99–1.04).

**Conclusions:**

In this large cohort study of individuals of European ancestry, gene scores for lipid fractions were not associated with incident AF.

## Introduction

Atrial fibrillation (AF) is the most common sustained cardiac arrhythmia, and is associated with increased risks of heart failure, stroke, and cardiovascular death,[1] including a 9-fold higher risk of mortality within the first four months after AF, compared to those without AF.[2] Some major risk factors for AF include age, white race, obesity, heart failure, coronary heart disease, left ventricular hypertrophy, and hypertension, along with certain lifestyle factors.[3–5] These predictors are similar to the risk factors for cardiovascular disease (CVD) in general, which often precede an AF diagnosis.[1]

High levels of total cholesterol, triglycerides and low-density lipoprotein cholesterol (LDLc) and low levels of high-density lipoprotein cholesterol (HDLc) have long been associated with CVD. The associations between lipid levels with incident AF have been inconsistent across observational studies.[6–11] However, several cohorts have reported associations between lower levels of LDLc and total cholesterol with incident AF, while others have reported no association.[6,8–11] In particular, the Atherosclerosis Risk in Communities (ARIC) study and the Women's Health Study both found inverse associations between LDLc and incident AF; hazard ratio (HR) and 95% confidence interval (CI) per 1 standard deviation increase = 0.90 (0.85–0.96) and HR (95% CI) comparing the top quintile to bottom quintile = 0.72 (0.56–0.92), respectively.[6,10] Table 1 depicts the previously reported associations between AF and blood lipids from observational cohorts, including 3 of the cohorts evaluated in the present analysis. Reasons for this inverse association are not clear, and it is not possible to determine if cholesterol has a direct causal effect on AF risk in observational studies.

**Table 1:** Previously discovered multivariable-adjusted cohort-specific Hazard Ratios and 95% Confidence Intervals of the risk of atrial fibrillation associated with blood lipids. Associations are per 1 standard deviation increase unless noted. ARIC, Atherosclerosis Risk in Communities; FHS, Framingham Heart Study; WHS, Women's Health Study; MESA, Multi-Ethnic Study of Atherosclerosis; CHS, Cardiovascular Health Study.

Cohort	Total Cholesterol	HDLc	LDLc	Triglycerides
ARIC [6]	0.89 (0.84–0.95)	0.97 (0.91–1.04)	0.90 (0.85–0.96)	1.00 (0.96–1.04)
FHS [7]	0.99 (0.87–1.12)	0.93 (0.81–1.08)	0.95 (0.82–1.09)	1.15 (1.02–1.30)
WHS[10]	0.76 (0.59–0.98) ^a^	1.07 (0.83–1.39) ^a^	0.72 (0.56–0.92) ^a^	0.83 (0.63–1.09) ^a^
MESA [7]	1.13 (0.98–1.30)	0.85 (0.72–1.00)	1.15 (0.99–1.35)	1.16 (1.02–1.33)
CHS [9]	0.86 (0.76–0.98)			
Watanabe *et al.[8]*		0.93 (0.85–1.02) ^b^	0.92 (0.88–0.96) ^b^	0.98 (0.96–1.00) ^b^
Iguchi *et al.[11]*	0.75 (0.58–0.96) ^c^			

^a^Comparing quintile 5 to quintile 1

^b^Per 10 mg/dL increase

^c^Odds ratio of total cholesterol > = 220

One approach to evaluate whether lipid levels are causally associated with AF risk is to perform a Mendelian randomization analysis, which uses genetic variants as an instrumental variable in determining association with an outcome independent of confounders.[12] Several genome-wide association studies (GWAS) have identified genes associated with blood lipoprotein levels, [13–15] including a meta-analysis of > 100,000 individuals of European ancestry by Teslovich and colleagues that identified 95 loci significantly associated with lipid phenotypes.[16] Data from that analysis have been used to create phenotype-specific lipid gene scores to estimate the effect of lipid genes on lipid levels.[17,18] We used these lipid gene scores as instrumental variables to examine the association between lipid levels and incident AF in 7 US and European prospective cohort studies participating in the AF Genetics (AFGen) consortium.[19]

## Methods

### Study Cohorts

Data from the following 7 cohorts was included: the Age, Gene/Environment Susceptibility—Reykjavik study (AGES), the Atherosclerosis Risk in Communities (ARIC) study, the Framingham Heart Study (FHS), the Malmö Diet and Cancer study (MDCS), 2 cohorts from the Rotterdam Study (RS-I and RS-II), and the Women's Genome Health Study (WGHS). A brief description of each participating cohort is provided below, with more information in the S1 File. Each cohort determined which examination to select as baseline, based on the availability of genetic and lipid data, covariates, date of DNA draw, and adequate follow-up time for the development of AF. Our analysis includes consenting participants with complete genetic data who were of white race. Participants were excluded if they had previous AF at baseline, had missing information of AF status at baseline, and those with missing covariates of interest. After applying exclusion criteria, the entire sample included 64,901 participants. Institutional Review Boards at the participating institutions approved the individual studies and study participants provided written informed consent.

#### Age, Gene/Environment Susceptibility Reykjavik Study

The original Reykjavik Study, conducted between 1967 and 1996, included ~ 19,000 men and women living in the greater Reykjavik area, born between 1907 and 1935.[20] Survivors of this study were invited to be part of AGES, which recruited 5764 men and women in 2002–2007, considered baseline years for this study. Of the survivors, 2953 met inclusion criteria and were considered for this analysis. Participant follow-up for this analysis was through 2012.

The AGES study was approved by the National Bioethics Committee in Iceland (VSN 00–063) as well as the Institutional Review Board of the Intramural Research Program of the National Institute on Ageing and the Data Protection Authority in Iceland.

#### Atherosclerosis Risk in Communities study

The ARIC study is a prospective cohort study of 15,792 men and women aged 45–64, recruited from four communities in the US (Washington County, MD; suburbs of Minneapolis, MN; Jackson, MS; Forsyth County, NC) in 1987–89.[21] Participants were mostly white in the Washington County and Minneapolis centers, only African-American in the Jackson center, and included both races in Forsyth County. After the initial assessment, study participants were examined four additional times (1990–92, 1993–95, 1996–98, 2011–13). Our analysis includes 8849 ARIC white participants that met inclusion criteria at baseline (1987–89). Follow-up for this analysis was through 2009. The University of Minnesota Institutional Review Board approved the present ARIC study, and all participants enlisted in the ARIC study have given their written informed consent.

#### Framingham Heart Study

In 1971–1975, the FHS Offspring cohort enrolled 5124 predominantly white men and women, offspring (and their spouses) from the Original FHS cohort (1948–1950) with follow-up examinations every 4 to 8 years. [22] The current analysis included 4126 participants of the FHS Offspring cohort that were free of AF, and were followed through 2011. The FHS was approved by the Institutional Review board of Boston University Medical center, and all participants enlisted in this study have given their written informed consent.

#### Malmö Diet and Cancer Study

The Malmö Diet and Cancer Study is a prospective study and recruited 45-64-year-old men and women in 1991–96, living in Malmö, Sweden, a city which then had a population of 230,000 inhabitants.[23] Baseline examinations were performed in 30,447 participants. For this study, 28,218 participants met inclusion criteria and were considered for this analysis. Follow-up was through 2009. MDCS was approved by the ethics committee of Lund University, Sweden and informed written consent was obtained from all participants in the study.

#### Rotterdam Study

The RS, a prospective population-based study aimed to assess the determinants of chronic conditions in the elderly, started in 1989 in Rotterdam.[24] At baseline, 7983 participants were included and in 2000, an additional 3011 participants were included. Participants have been continuously followed and were reexamined approximately every 4–5 years. The present analysis included participants that met inclusion criteria: 4560 study participants examined in 1989–93, and 1689 examined in 2000–2001. Follow-up for both cohorts was through 2011. RS was approved by the medical ethics committee of Erasmus University, and written consent was obtained from all participants.

#### Women's Genome Health Study

WGHS is an ongoing prospective cohort GWAS that derives from the NIH-funded Women’s Health Study (WHS) and includes more than 25,000 initially healthy women aged 45 years and older, who have already been followed since 1992–95 for the development of common disorders such as CVD.[25] Our analysis includes 14,056 eligible females that were followed through 2011. Informed consent was obtained from all participants in the WGHS, and the Brigham and Women's Hospital Institutional Review Board for Human Subjects Research approved the study protocol.

### Ascertainment of atrial fibrillation

AF cases in all cohorts were ascertained using a variety of cohort-specific methods, including study physician-adjudicated cases, study electrocardiograms, hospitalization discharge diagnosis codes, or death certificates (ICD-9-CM 427.3, 427.31 or 427.32, or ICD-10 I48 in any position).[1,3,9,26] Further details of AF ascertainment in each cohort are available in the S1 File. In this analysis, the incidence date of AF was defined as the date of the first ECG showing AF, date of diagnosis within the medical record, the first hospital discharge coded as AF, or when AF was listed as a cause of death, whichever occurred earlier.

### Assessment of lipid gene scores

In all studies, genotyping was performed on baseline samples. Genotyping and quality control methods are described elsewhere for each study, and the S1 File contain a brief overview for genotyping information (Table A in S1 File). [19,27–29] Of note, MDCS had slightly different genotyping methods, and thus their lipid gene scores are marginally lower compared to the other cohorts.[27] MDCS did not have available SNPs for total cholesterol, and any other differences in scores are noted in Table B in S1 File.

To create the lipid gene scores, we used SNPs identified in the Teslovich et al. GWAS. [16] Separate effect size-based gene scores were created for each lipid phenotype (HDLc, LDLc, total cholesterol and triglycerides). More detailed creation of the scores is described elsewhere. [17] Briefly, for each individual, the number of unfavorable alleles (0, 1 or 2) for each SNP was multiplied by the absolute value of the additive effect size published in the Teslovich et al. GWAS.[16] The product was summed across each individual SNP and then rescaled by dividing that sum by the average effect size. Lastly, cohorts divided their phenotype-specific gene score by 1 standard deviation. A second gene score was created in the same manner for HDLc and LDLc including only SNPs exclusively associated with each specific cholesterol fraction.[18] This non-pleiotropic score was then used to assess potential causal effects of each fraction without including indirect effects through other cholesterol fractions, as recently described. [18] The final scores included 47 SNPs for HDLc, 37 SNPs for LDLc, 52 SNPs for total cholesterol, and 32 SNPs for triglycerides (listed in Table B in S1 File). The final scores for MDCS included 41 SNPs for HDLc, 32 SNPs for LDLc, and 27 SNPs for triglycerides. The non-pleiotropic score for HDLc included 14 SNPs and LDLc included 13 SNPs.

### Measurement of other covariates

All study cohorts collected information on anthropometric measures, blood pressure, blood lipids, fasting glucose, as well as assessment of prior cardiovascular disease and medication use. Details on measurement methods are provided in the S1File. Protocols for variable ascertainment and definitions of cardiovascular risk factors were comparable across cohorts.

### Statistical analysis

We estimated the association of a 1 standard deviation (cohort-specific) increase in each lipid gene score with the incidence of AF using Cox proportional hazards models with time to AF as the main outcome variable. Follow-up time was defined as the time elapsed between baseline and date of AF incidence, death, lost to follow-up or until the end of available data, whichever came earlier. Models were adjusted for baseline covariates including age, sex, study site (for ARIC), cohort (for FHS), education (high school graduate vs. not; N/A in FHS), height (continuous), smoking (current vs. not), body mass index (continuous), systolic blood pressure (continuous), diastolic blood pressure (continuous), use of antihypertensive medications, diabetes mellitus (dichotomous), electrocardiograph left ventricular hypertrophy, previous stroke, previous heart failure and previous coronary heart disease. An additional model was performed also adjusting for continuous lipid levels (N/A in MDCS) and lipid medication use. Cohorts missing information on lipid levels, left ventricular hypertrophy, previous stroke, previous heart failure, or previous coronary heart disease only adjusted for the available variables. With 65,000 participants, 8% having AF, a trait variance of 5% explained by lipid genes, and an odds ratio of 0.80 for the association between blood lipids and AF incidence, this instrument had 88% power to detect an association between lipid gene scores and AF in this Mendelian randomization study.[30] Finally, results were meta-analyzed for each specific lipid gene score using a fixed-effects model. All statistical analyses were performed with SAS v 9.2 (SAS Inc, Cary, NC) or STATA (v.13, STATA Corp).

## Results

Selected baseline characteristics for cohort participants are shown in Table 2. There were a total of 64,901 participants in all cohorts combined. The cohorts had a higher prevalence of women than men. The participants’ mean ages at baseline ranged from 54 years in ARIC and WGHS to 76 years in AGES. The gene scores were similar across cohorts, with the MDCS score being slightly lower, due to unavailability of some components of the score. During a mean follow-up of 12 years, 5434 (8.4%) incident AF cases were identified. Cumulative risk ranged between 4% in WGHS and 14% in AGES, ARIC, and FHS. The study-specific risk of incident AF cases depended on 1) age at baseline, with older cohorts having a higher incidence; 2) the amount of follow-up time in the study, since more cases would be captured in a longer time frame; and 3) the gender of the participants, with women having a lower risk of AF when compared to men. Thus, higher cumulative risk of AF was observed in studies with the long follow-up, like ARIC, and older age at baseline, like AGES, while studies recruiting younger participants, WGHS, and with shorter follow-up (Rotterdam Study-II) reported lower risks.

**Table 2:** Baseline characteristics of study participants by cohort. Values correspond to N (%) or mean (standard deviation). The standard deviation of each gene score in each cohort is 1 due to the method of standardization used.—AGES, indicates the Age, Gene/Environment Susceptibility—Reykjavik study; ARIC, Atherosclerosis Risk in Communities; FHS, Framingham Heart Study; MDCS, Malmö Diet and Cancer study; RS, Rotterdam Study; WGHS, Women's Genome Health Study; SBP, systolic blood pressure; DBP, diastolic blood pressure; HDLc, high density lipoprotein cholesterol; LDLc, low density lipoprotein cholesterol; LVH, left ventricular hypertrophy; NA, not available, * not fasting.

	AGES (n = 2953)	ARIC (n = 8849)	FHS (n = 4126)	MDCS (n = 28,218)	RS-I (n = 4560)	RS-II (n = 1689)	WGHS (n = 14506)
Age, years	76 (5)	54 (6)	64 (13)	58 (8)	68 (8)	65 (8)	54 (7)
Men	1341 (42%)	4117 (47%)	1795 (44%)	11135 (40%)	1860 (41%)	774 (46%)	0
Height, cm	167 (9)	169 (10)	166 (10)	169 (9)	167 (9)	169 (9)	164 (6)
Current cigarette smoking	404 (13%)	2151 (24%)	613 (15%)	8076 (29%)	1074 (24%)	375 (22%)	1653 (11%)
Body mass index, kg/m²	27 (4)	27 (5)	28 (5)	26 (4)	26 (4)	27 (4)	26 (5)
SBP, mmHg	143 (20)	118 (17)	130 (20)	141 (20)	139 (22)	143 (21)	124 (14)
DBP, mmHg	74 (10)	72 (10)	74 (10)	86 (10)	74 (11)	79 (11)	77 (9)
Hypertensive medication	2036 (64%)	2184 (25%)	1453 (35%)	4786 (17%)	1903 (42%)	452 (27%)	1886 (13%)
Diabetes mellitus	366 (11%)	691 (8%)	327 (8%)	810 (2.9)	437 (10%)	179 (11%)	320 (2%)
HDLc < 40 mg/dL	261 (8%)	2510 (28%)	899 (23%)	NA	4421 (97%)*	1656 (98%)*	2342 (16%)
LDLc ≥160mg/dL	846 (27%)	2240 (25%)	450 (14%)	NA	NA	NA	2096 (14%)
Total cholesterol, ≥ 240 mg/dL	919 (28%)	2070 (23%)	620 (16%)	NA	2 (0%)*	0 (0%)	3219 (22%)
Triglycerides, ≥ 200 mg/dL	180 (6%)	1191 (13%)	665 (18%)	NA	NA	1 (0%)	2317 (16%)
LVH	NA	74 (1%)	41 (1%)	NA	174 (4%)	36 (2%)	NA
Previous stroke	NA	125 (2%)	96 (2%)	290 (1%)	102 (2%)	57 (3%)	NA
Previous heart failure	97 (3%)	309 (3%)	53 (1%)	57 (0.2%)	103 (2%)	37 (2%)	NA
Previous coronary heart disease	NA	418 (5%)	284 (7%)	538 (2%)	579 (13%)	85 (5%)	NA
Lipid lowering medication use	724 (23%)	295 (3%)	625 (15%)	778 (3%)	101 (2%)	203 (12%)	513 (4%)
HDLc gene score	9.1	9.3	10.1	8.6	10.0	10.0	9.9
LDLc gene score	9.7	9.5	9.5	8.4	9.4	9.4	9.4
Total cholesterol gene score	12.7	12.7	12.8	NA	12.6	12.6	12.3
Triglyceride gene score	9.4	9.6	9.7	8.4	9.6	9.5	9.4
# Atrial fibrillation cases	422 (14%)	1207 (14%)	565 (14%)	2087 (7%)	571 (13%)	78 (5%)	504 (4%)
Mean follow-up time, years (SD)	7.3 (3)	18.6 (5)	9.7 (4)	13.8 (4)	13.2 (6)	6.6 (1)	15.6 (3)
Baseline years	2002–2007	1987–1989	1987–2007	1991–96	1989–93	2000–01	1992–1995
End of follow-up	2012	2009	2011	2009	2011	2011	2011

**Fig 1:**
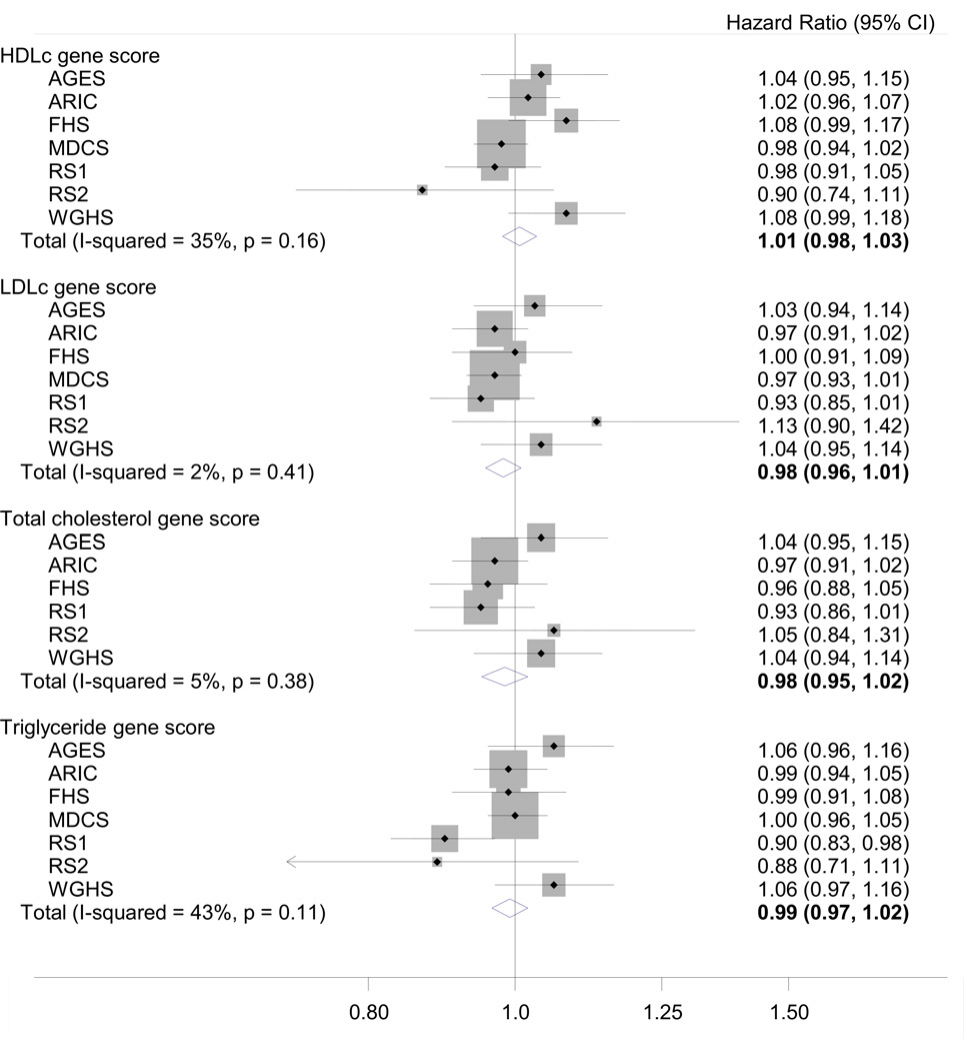
Meta-analysis of the association of lipid gene scores, and atrial fibrillation. Association is adjusted for age, sex, center, education, height, smoking status, body mass index, systolic blood pressure, diastolic blood pressure, use of antihypertensive medication, diabetes, left ventricular hypertrophy, previous stroke, previous coronary heart disease, and previous heart failure

After adjustment for potential confounders and intermediates there was no significant association between any of the lipid gene scores with incident AF in the individual cohorts (Table 3). When models were adjusted for lipid levels and lipid medication use, there was little change in the results, and associations remained similar across all cohorts. A meta-analysis of findings across all cohorts showed no significant association between any of the lipid gene scores with incident AF (Fig 1). HR and 95% CI for AF with a 1-SD increase in HDLc gene score was 1.01 (0.98–1.03); for LDLc, 0.98 (0.96–1.01); total cholesterol, 0.98 (0.95–1.02) and triglycerides, 0.99 (0.97–1.02). No causal estimate for the effect of concentrations of blood lipids on AF risk was calculated due to the null results.

**Table 3:** Multivariable adjusted Hazard Ratios (95% confidence interval) of Atrial Fibrillation of a 1 Standard Deviation Increase in Lipid Gene Score, by Cohort. HR, Hazard Ratio; CI, Confidence interval; NA, not available. -AGES, indicates the Age, Gene/Environment Susceptibility—Reykjavik study; ARIC, Atherosclerosis Risk in Communities; FHS, Framingham Heart Study; MDCS, Malmö Diet and Cancer study; RS, Rotterdam Study; WGHS, Women's Genome Health Study. Model 1: Cox proportional hazard model adjusted for age, sex and study center, if appropriate. Model 2: Model 1 + education, height, smoking status, body mass index, systolic blood pressure, diastolic blood pressure, use of antihypertensive medication, diabetes, left ventricular hypertrophy, previous stroke, previous coronary heart disease and previous heart failure. Model 3: Model 2 + adjusted for continuous lipid levels and lipid medication use.

	AGES	ARIC	FHS	MDCS	RS-I	RS-II	WGHS
HDLc gene score							
Model 1: HR (95% CI)	1.04 (0.94–1.14)	1.01 (0.95–1.06)	1.06(0.98–1.15)	0.97 (0.93–1.01)	0.99 (0.92–1.07)	0.90 (0.74–1.10)	1.08 (0.99–1.18)
Model 2: HR (95% CI)	1.04 (0.95–1.15)	1.02 (0.96–1.07)	1.08(0.99–1.17)	0.98 (0.94–1.02)	0.98 (0.91–1.05)	0.90 (0.74–1.11)	1.08 (0.99–1.18)
Model 3: HR (95% CI)	1.02 (0.92–1.13)	1.02 (0.96–1.08)	1.05(0.94–1.16)	NA	0.98 (0.91–1.06)	0.92 (0.74–1.13)	1.10 (1.01–1.21)
LDLc gene score							
Model 1: HR (95% CI)	1.03 (0.93–1.14)	0.97 (0.91–1.02)	1.00(0.91–1.09)	0.97 (0.93–1.02)	0.92 (0.85–1.00)	1.07 (0.86–1.34)	1.04 (0.95–1.13)
Model 2: HR (95% CI)	1.03 (0.94–1.14)	0.97 (0.91–1.02)	1.00(0.91–1.09)	0.97 (0.93–1.01)	0.93 (0.85–1.01)	1.13 (0.90–1.42)	1.04 (0.96–1.14)
Model 3: HR (95% CI)	1.04 (0.94–1.15)	0.97 (0.91–1.02)	1.02(0.91–1.15)	NA	0.95 (0.87–1.04)	1.19 (0.93–1.51)	1.08 (0.98–1.18)
Total cholesterol gene score							
Model 1: HR (95% CI)	1.04 (0.94–1.14)	0.97 (0.91–1.02)	0.96(0.88–1.05)	NA	0.92 (0.85–1.00)	1.01 (0.81–1.26)	1.04 (0.95–1.13)
Model 2: HR (95% CI)	1.04 (0.95–1.15)	0.97 (0.91–1.02)	0.96(0.88–1.05)	NA	0.93 (0.86–1.01)	1.05 (0.84–1.31)	1.04 (0.96–1.14)
Model 3: HR (95% CI)	1.06 (0.96–1.17)	0.97 (0.91–1.02)	0.97(0.87–1.09)	NA	0.96 (0.88–1.04)	1.09 (0.86–1.37)	1.07 (0.98–1.17)
Triglyceride gene score							
Model 1: HR (95% CI)	1.05 (0.96–1.16)	0.99 (0.93–1.04)	0.99(0.91–1.08)	0.99 (0.95–1.04)	0.91 (0.83–0.98)	0.89 (0.72–1.11)	1.06 (0.98–1.16)
Model 2: HR (95% CI)	1.06 (0.96–1.16)	0.99 (0.94–1.05)	0.99(0.91–1.08)	1.00 (0.96–1.05)	0.90 (0.83–0.98)	0.88 (0.71–1.11)	1.06 (0.97–1.16)
Model 3: HR (95% CI)	1.06 (0.96–1.18)	0.99 (0.94–1.05)	0.98(0.88–1.09)	NA	0.91 (0.84–1.00)	0.91 (0.72–1.15)	1.07 (0.98–1.17)

**Fig 2:**
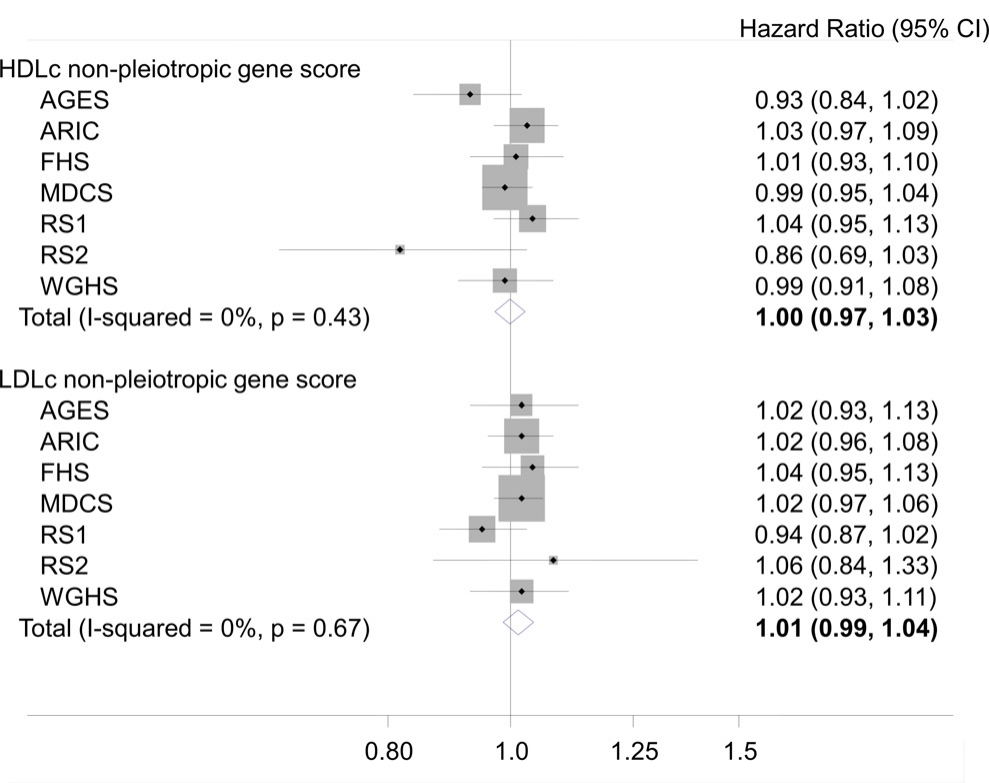
Meta-analysis of the association of non-pleiotropic HDLc and LDLc gene scores, and atrial fibrillation. Association is adjusted for age, sex, center, education, height, smoking status, body mass index, systolic blood pressure, diastolic blood pressure, use of antihypertensive medication, diabetes, left ventricular hypertrophy, previous stroke, previous coronary heart disease, and previous heart failure

Narrowing the HDLc and LDLc gene scores to non-pleiotropic genes did not result in any significant associations between incident AF and an increase in scores (Table 4). Fig 2 shows the meta-analysis for the non-pleiotropic scores. Overall, the association [HR (95% CI)] between HDLc and AF was 1.00 (0.97–1.03) and for LDLc 1.01 (0.99–1.04).

**Table 4:** Multivariable adjusted Hazard Ratios (95% confidence interval) of Atrial Fibrillation of a 1 Standard Deviation Increase in Non-pleiotropic Lipid Gene Score, by Cohort. HR, Hazard Ratio; CI, Confidence interval; NA, not available. AGES, indicates the Age, Gene/Environment Susceptibility—Reykjavik study; ARIC, Atherosclerosis Risk in Communities; FHS, Framingham Heart Study; MDCS, Malmö Diet and Cancer study; RS, Rotterdam Study; WGHS, Women's Genome Health Study. Model 1: Cox proportional hazard model adjusted for age, sex and study center, if appropriate. Model 2: Model 1 + education, height, smoking status, body mass index, systolic blood pressure, diastolic blood pressure, use of antihypertensive medication, diabetes, left ventricular hypertrophy, previous stroke, previous coronary heart disease and previous heart failure. Model 3: Model 2 + adjusted for continuous lipid levels and lipid medication use.

	AGES	ARIC	FHS	MDCS	RS-I	RS-II	WGHS
HDLc non-pleiotropic gene score							
Model 1: HR (95% CI)	0.93 (0.85–1.03)	1.02 (0.97–1.08)	1.01(0.94–1.10)	1.00 (0.96–1.05)	1.05 (0.96–1.14)	0.84 (0.68–1.04)	0.99 (0.90–1.08)
Model 2: HR (95% CI)	0.93 (0.84–1.02)	1.03 (0.97–1.09)	1.01(0.93–1.10)	0.99 (0.95–1.04)	1.04 (0.95–1.13)	0.86 (0.69–1.08)	0.99 (0.91–1.08)
Model 3: HR (95% CI)	0.93 (0.84–1.03)	1.03 (0.97–1.09)	1.01(0.91–1.12)	1.06 (0.96–1.17)	1.04 (0.95–1.13)	0.85 (0.68–1.07)	0.98 (0.90–1.08)
LDLc non-pleiotropic gene score							
Model 1: HR (95% CI)	1.02 (0.93–1.13)	1.02 (0.96–1.08)	1.05(0.96–1.14)	1.02 (0.98–1.07)	0.93 (0.86–1.01)	1.03 (0.82–1.30)	1.02 (0.93–1.11)
Model 2: HR (95% CI)	1.02 (0.93–1.13)	1.02 (0.96–1.08)	1.04(0.95–1.13)	1.02 (0.97–1.06)	0.94 (0.87–1.02)	1.06 (0.84–1.33)	1.02 (0.93–1.11)
Model 3: HR (95% CI)	1.02 (0.92–1.12)	1.02 (0.96–1.08)	1.03(0.93–1.15)	1.04 (0.93–1.15)	0.96 (0.88–1.04)	1.06 (0.84–1.35)	1.03 (0.94–1.13)

## Discussion

In our prospective, multi-cohort study including 64,901 white participants from 7 large cohorts, with 5434 AF cases, we found a consistent lack of association between the lipid genetic scores and incidence of AF. Similarly, narrowing the genetic score to genes more specific for HDLc or LDLc levels showed no independent associations between non-pleiotropic genetic scores and risk for AF. These associations were adjusted for lifestyle factors, clinical factors, and cardiovascular disease. The results of our Mendelian randomization approach, in which we used lipid gene scores as instrumental variables, do not support a casual association between blood lipid levels and risk of AF, assuming that these genetic variants could only cause AF through mediating effects of these lipids.

The paradoxical association of LDLc with incident AF, though inconsistent, has been seen in a number of studies, and remains unexplained (Table 1).[6,8–11] Some proposed mechanisms include cholesterol-mediated changes in the distribution of atrial ion channels,[31] unmeasured confounding by hyperthyroidism,[32] malnutrition, fluid imbalances, or by other confounders including natriuretic peptides.[7] A recent analysis of the Women’s Health Study also reported an inverse association between blood LDLc and AF risk, and this association was mainly driven by cholesterol-poor small LDL particles and small VLDL particles. No association was observed between cholesterol-rich LDL particles and AF, suggesting that risk for AF is probably not mediated through a direct effect of cholesterol.[10] If small LDL particles are truly responsible for the inverse association of LDL cholesterol and AF, one explanation for the absence of association between LDLc gene scores and AF in our study is that the cholesterol genes that have been discovered and included in this lipid gene score could be associated with the cholesterol component of lipoprotein particles. This potential explanation may be explored further as more specific genetic variants affecting lipoprotein particle size are discovered.

The associations between HDLc, triglycerides and incident AF have been inconsistent, with some studies reporting an increased risk of AF in those with low HDLc levels or high triglyceride levels, and other studies finding no association between those lipid levels and AF. [6–11] These inconsistencies remain unexplained and our findings would suggest that associations between AF and low HDLc are not mediated by known polymorphisms associated with HDL levels. It is possible that the associations observed in some studies may be due to residual confounding by other cardiovascular or metabolic risk factors, since low HDLc and high triglycerides can be part of the metabolic syndrome, which has also been associated with AF risk. [33]

### Strengths and limitations

A few limitations should be noted. First, the studied cohorts recruited different populations across diverse settings using a variety of methods for measurement of covariates and ascertainment of AF. Despite these differences, no evidence of heterogeneity was observed in the meta-analysis of cohort-specific results. Second, some cohorts mostly relied on electrocardiograms performed at study visits and hospital discharge codes, leading to the potential for misclassification of AF, though validation in ARIC and other populations have demonstrated adequate specificity with positive predictive value of ~90%.[3,9] Third, asymptomatic AF and AF managed exclusively in an outpatient setting were not consistently identified. Fourth, some SNPs in the GWAS score were imputed, and the genes scores only explain a portion of the variation seen in lipid levels. [17] For example, in ARIC, Lutsey et al. found that each phenotype-specific gene score explained 1.6% of the variance in HDLc levels, 6% of the variance in LDLc and triglycerides, and 6.8% of the variance in total cholesterol.[17] Therefore, it is unlikely that all relevant lipid genes have been identified. A more recent list of lipid SNPs in 157 loci (62 new loci in addition to the 95 used in the Teslovich study) was published after completion of data analysis in all cohorts in our study. [34] We decided not to rerun our analysis for logistical reasons, after considering the effect of the 62 new loci on lipid levels was generally smaller than in the earlier GWAS (1.6–2.6% variance explained vs. 10–12% variance in the Teslovich loci), and therefore, with our current analysis, we likely would have found an association if one existed. Finally, our results may not be generalizable to nonwhites. Despite these limitations, our study has important strengths including a large sample size, long follow-up, a large number of incident AF events, and an extensive list of measured covariates.

## Conclusions

In conclusion, in our sample of approximately 65,000 white participants, lipid gene scores for HDLc, LDLc, total cholesterol, and triglycerides were not associated with incident AF, meaning we did not find a direct relationship between lipid levels and the risk of AF.

## Supporting Information

S1 File Supporting Information Supplemental methods, Tables A and B in S1 File. **Table A in S1 File.** Select details regarding study samples and genotyping. **Table B in S1 File.** SNPs originally identified in the Teslovich *et al*. GWAS included in our lipid gene scores, along with their unfavorable allele and effect size.
(DOCX)
