# Supplementary material for: Association of Lipid-Related Genetic Variants with the Incidence of Atrial Fibrillation: The AFGen Consortium
Source: PLoS One. 2016 Mar 21;11(3):e0151932. doi: 10.1371/journal.pone.0151932 (PMC4801208; doi:10.1371/journal.pone.0151932)
Supplement: S1 File — Table A in S1 File. Select details regarding study samples and genotyping. Table B in S1 File. SNPs originally identified in the Teslovich et al. GWAS included in our lipid gene scores, along with their unfavorable allele and effect size. (DOCX) [file pone.0151932.s001.docx]

**S1 File. Supporting Information**

**Supplemental methods**

**Atrial fibrillation ascertainment**

**AGES**

Atrial fibrillation (AF) or atrial flutter was diagnosed from Minnesota-coded electrocardiogram (ECG) recorded at AGES study exams and from ICD-9 427.3 or ICD-10 I48 codes from hospital discharges in the National Hospital of Iceland database through April 2010.[[1](#_ENREF_1)]

**ARIC**

AF in ARIC was identified from 3 sources: ECG conducted at each study visit, hospital discharge codes (ICD-9CM 427.31, AF, or 427.32, atrial flutter), and death certificates (ICD-9 427.3 or ICD-10 I48). AF cases occurring during the same hospitalization as open cardiac surgery were not included as events.[[2](#_ENREF_2),[3](#_ENREF_3)]

**FHS**

Participants were classified as having AF if atrial fibrillation or atrial flutter was present on an ECG derived from a Framingham Study clinic tracing, on an ECG during an encounter with an external clinician, or by Holter monitoring, or if it was noted in hospital records. All incident AF cases were reviewed and adjudicated by one of two Framingham cardiologists.[[4](#_ENREF_4),[5](#_ENREF_5)]

**MDCS**

Prevalent or incident AF in the Malmö Diet and Cancer was identified from national registers as previously described. [[6](#_ENREF_6)] Briefly, AF was ascertained by linkage of Swedish personal identification numbers with hospitalization and death registers using ICD diagnosis codes 427.92 for ICD-8, 427D for ICD-9 and I48 for ICD-10. AF was defined as either a diagnosis of AF or atrial flutter.

**RS**

AF cases were ascertained at baseline and during follow-up as described previously.[[7](#_ENREF_7)] Briefly, ECGs were recorded and stored digitally, and analyzed by the Modular ECG Analysis System. Two research physicians and a cardiologist verified AF diagnoses. Additional information was obtained from general practitioner records, from outpatient clinics, and from a national database of hospitalizations, which records all hospitalization discharge diagnoses occurring in the Netherlands. AF cases occurring during a serious disease resulting in death, or during myocardial infarction or cardiac operative procedures who recovered during the hospital admission were not included.

**WGHS**

Women were asked to report diagnoses of AF at baseline, 48 months, and then annually thereafter. Beginning on September 19, 2006, women enrolled in the continued observational follow-up who reported an incident AF event on at least one yearly questionnaire were sent an additional questionnaire to confirm the episode and to collect additional information. They were also asked for permission to review their medical records, particularly available ECGs, rhythm strips, 24-hour ECGs, and information on cardiac structure and function. For all deceased participants who reported AF during the trial and extended follow-up period, family members were contacted to obtain consent and additional relevant information. An end-point committee of physicians reviewed medical records for reported events according to predefined criteria. An AF event was confirmed if there was ECG evidence, or if a medical report clearly indicated a personal history of AF. The earliest date in the medical records when documentation was believed to have occurred was set as the date of onset of AF. Only confirmed events are included in this analysis. [[8](#_ENREF_8)]

**Assessment of covariates**

**AGES**

Standard examination protocols and questionnaires were completed in the AGES study. Clinic visits included anthropometry, blood pressure measurement (defined as the mean value of two consecutive blood pressure measurements), electrocardiogram, and measures of different physical and cognitive function domains. Blood samples were drawn after overnight fasting. Total cholesterol, HDLc, triglycerides, and glucose were analyzed on a Hitachi 912, using reagents from Roche Diagnostics and following the manufacturer's instructions. LDLc was calculated using the Friedewald equation. Diabetes was defined from a self-report of a physician diagnosis, use of oral hypoglycemic agents or insulin, or a fasting blood glucose ≥126 mg/dL. Diagnoses of myocardial infarction and heart failure were based on hospital discharge records.[[1](#_ENREF_1)]

**ARIC**

Study participants were asked to fast before the clinic visit, during which a blood sample was obtained and a physical examination performed. Race, education and smoking status were determined by participant self-report. Blood collection and processing techniques for the ARIC study have been previously described.[[9](#_ENREF_9)] Enzymatic methods were used to measure total cholesterol and triglycerides. HDLc was measured enzymatically after dextran sulfate- Mg2+ precipitation of other lipoproteins. LDLc was estimated using the Friedewald formula for subjects with triglycerides levels <400 mg/dl. Body mass index (BMI) was calculated as weight (in kilograms) divided by height (in meters) squared. Blood pressure was measured 3 times with the subject in the sitting position after 5 minutes of rest using a random-zero sphygmomanometer, and the last 2 measurements were averaged. Participants were asked to bring all medications with them to the clinic visits. A prescription bottle or self-report was used to determine cholesterol and blood pressure medication use. A 12-lead ECG at rest was used to define the presence of left ventricular hypertrophy (LVH). ECG- diagnosed LVH was considered present if the Cornell voltage was >28 mm in men or >22 mm in women. Diabetes was categorized as a fasting glucose of ≥126 mg/dl or nonfasting glucose level of ≥200 mg/dl, a self-reported physician diagnosis of diabetes, or currently taking medication for diabetes. Prevalent coronary heart disease at baseline included a history of myocardial infarction, myocardial infarction adjudicated from the baseline ECG, or a history of coronary bypass or angioplasty. Prevalent heart failure was defined as a previous hospitalization for heart failure. [[9](#_ENREF_9)]

**FHS**

In the FHS, medication, alcohol use, and smoking were ascertained by self-report. Current smoking was defined as regular use of one or more cigarettes/day within the year prior to the Framingham clinic visit. Glucose and lipids were measured after an overnight fast in the Framingham Study laboratory. Diabetes was diagnosed as fasting glucose ≥126 mg/dL, or use of hypoglycemic medications. Blood pressure was determined by FHS physicians in the seated participants as the average of two systolic and diastolic blood pressure measurements in mm Hg. Cardiovascular events were adjudicated by a panel of 3 physicians, examining participant hospitalization and outpatient records. Heart failure was diagnosed based on major and minor clinical criteria that have been used for all heart failure cases of Framingham Heart Study participants. Myocardial infarction was diagnosed based on the presence of clinical history, electrocardiographic signs, and biomarkers. ECG-based LVH was considered present when a participant met voltage criteria for left ventricular hypertrophy with lateral repolarization changes.[[5](#_ENREF_5),[10](#_ENREF_10),[11](#_ENREF_11)]

**MDCS**

In the Malmö Diet and Cancer study (MDCS), individuals underwent anthropometric measurements, blood pressure, and filled out a questionnaire on health, lifestyle, socioeconomic factors and medications. Smoking was defined as self-reported regular smoking or smoking cessation within the last year. Diabetes mellitus was defined as a self-reported physician’s diagnosis or use of anti-diabetic medications, or from one of five nation-wide or regional registers. Hypertension was defined as use of antihypertensive medication or blood pressure ≥140/90 mmHg. Cholesterol measurements were performed in a random subset of around 5300 participants. Levels of LDLc were calculated according to the Friedewald formula, in those with triglycerides <400 mg/dL. Because of the limited subset of participants with measured lipid levels, MDCS did not run the model which adjusted for continuous lipid levels. Cardiovascular events were identified through ICD codes.[[12](#_ENREF_12)]

**RS**

Information on current health status, medical history, and smoking was obtained using a computerized questionnaire. Participants were classified as current or non-smokers. BMI was calculated as weight in kilograms divided by the square of height in meters. Education level was based on interview data and classified according to whether the participant graduated high school. Blood pressure was measured twice at the right upper arm with a random zero mercury sphygmomanometer in the sitting position. Systolic and diastolic blood pressures were calculated as the average of the two consecutive measurements. History of stroke at baseline was assessed during the baseline interview and verified by reviewing medical records. A history of coronary heart disease was defined as a history of myocardial infarction (based on symptoms, ECG measurements and enzyme markers) or a percutaneous or surgical coronary revascularization procedure according to the medical files. Diabetes mellitus was defined as the use of blood glucose lowering medication, a fasting serum glucose level above 124 mg/dL, or a non-fasting serum glucose level of 198 mg/dL or more. Diagnosis of heart failure was based on a score of heart failure symptoms, on medication prescribed with the indication of heart failure, on hospital discharge diagnoses, and on the information available in general practitioner files. Left ventricular hypertrophy was determined on ECGs. Antihypertensive medication use was determined using the interview data on baseline. Blood samples were drawn by venipuncture, and serum total cholesterol and HDL cholesterol were measured with an automated enzymatic method.[[13](#_ENREF_13)] For RS-I baseline lipid levels were non-fasting.

**WGHS**

The Women’s Genome Health Study (WGHS) is a prospective cohort comprised of over 25,000 initially healthy female health professionals enrolled in the Women’s Health Study, which began in 1993. All participants in WGHS provided baseline blood samples and extensive survey data.[[8](#_ENREF_8)] Cardiovascular events are extracted from medical records, questionnaires, and death certificates. Total cholesterol and HDLc were measured enzymatically and analyzed on a Hitachi 911 autoanalyzer (Roche Diagnostics). LDLc was determined directly (Genzyme). Triglycerides were measured enzymatically, with correction for endogenous glycerol, using a Hitachi 917 analyzer and reagents and calibrators from Roche Diagnostics.

**Table A.** Select details regarding study samples and genotyping

|  | **AGES** | **ARIC** | **FHS** | **MDCS** | **RS-I** | **RS-2** | **WGHS** |
| --- | --- | --- | --- | --- | --- | --- | --- |
| **Study** | Age, Gene/ Environment Susceptibility Study | Atherosclerosis Risk in Communities Study | Framingham Heart Study | Malmo Diet and Cancer study | Rotterdam Study | | Women’s Genome Health Study |
| **Array** | Illumina  HumanCNV370-Duo BeadChip | Affymetrix 6.0 | Affymetrix Gene Chip® 500K Array Set & 50K Human  Gene Focused Panel | Sequenom MassArray or ABI 7900HT | Illumina Infinium  HumanHap550-  chip v3.0 | | Illumina HumanHap300 Duo+ |
| **Calling Algorithm** | BeadStudio | Birdseed | BRLMM | MassArray Typer 4.0 or SDS 2.3 | BeadStudio | | BeadStudio |
| **Per SNP Call rate** | <98% | <95% | <97% |  | <98% | | <90% |
| **HWE p-value** | <10^-5^ | <10^-6^ | <10^-6^ | <0.05 | <10^-6^ | | <10^-6^ |
| **Excess heterozygosity** | NA | NA | subject hetero-zygosity >5 SD away from the mean | NA | >0.336; n=21 | | ND |
| **MAF** | <1% | <1% | <1% | <1% | <1% | | <1% |
| **Number of SNPs used for imputation** | 308,340 | 602,642 | 385,958 | - | 530,683 | | 331959 |
| **Imputation software** | Mach1 v 1.0.16 | Mach1 v 1.0.16 | Mach1 v 1.0.15 | - | Mach1 v 1.0.15 | | Mach1 v. 1.0.16  HapMap II CEU r22 |
| **Imputation Backbone / NCBI Build** | Build 36 | Build 36 | Build 36 | - | Build 36 | | Build 36 |
| **SNP position from NCBI build** | Build 36 | Build 36 | Build 36 | - | Build 36 | | Build 36 |
| **GWAS Statistical Analysis** | ProbABEL, R | ProbABEL, PLINK, R | R packages kinship, gee, coxpH | - | Mach2QTL GenABEL + PLINK, R, GRIMP | | ProbABEL, R |
| **Inflation factor (λ)** | I: 1.005  P:1.062 | 1.007 | I:1.017  P:1.038 | - | I:1.035  P:1.024 | | 1.017 |

I, incident; NA, not available; ND, not done; P, prevalent; BRLMM, denotes the Bayesian Robust Linear Modeling.

PLINK, http://pngu.mgh.harvard.edu/purcell/PLINK/

Eigenstrat, http://genepath.med.harvard.edu/~reich/Software.htm

MACH, http://www.sph.umich.edu/csg/abecasis/MaCH/index.html

BIMBAM, http://stephenslab.uchicago.edu/software.html

**Table B.** SNPs originally identified in the Teslovich *et al.* GWAS included in our lipid gene scores, along with their unfavorable allele and effect size.

| Trait | SNP | Major Allele | Minor Allele | Effect Size | Unfavorable Allele | MDCS differences |
| --- | --- | --- | --- | --- | --- | --- |
| HDL | rs4660293 | A | G | -0.48 | G |  |
|  | **rs1689800** | A | G | -0.47 | G |  |
|  | rs4846914 | A | G | -0.61 | G |  |
|  | rs1042034 | T | C | 0.9 | T |  |
|  | rs12328675 | T | C | 0.68 | T |  |
|  | rs1515100 | A | C | 0.46 | A |  |
|  | **rs13107325** | C | T | -0.84 | T |  |
|  | rs6450176 | G | A | -0.49 | A |  |
|  | rs2814944 | G | A | -0.49 | A |  |
|  | rs605066 | T | C | -0.39 | C |  |
|  | rs1084651 | G | A | -0.56 | A | NA |
|  | rs17145738 | C | T | 0.57 | C |  |
|  | rs4731702 | C | T | 0.59 | C | NA |
|  | rs9987289 | G | A | -1.21 | A |  |
|  | rs12678919 | A | G | 2.25 | A |  |
|  | **rs2293889** | G | T | -0.44 | T |  |
|  | rs10808546 | C | T | 0.61 | C | Proxy, rs2954029 |
|  | rs643531 | A | C | -0.72 | C |  |
|  | rs1883025 | C | T | -0.94 | T |  |
|  | **rs2923084** | A | G | -0.41 | G |  |
|  | rs3136441 | T | C | 0.78 | T |  |
|  | rs174601 | C | T | -0.73 | T | Proxy, rs174546 |
|  | rs964184 | C | G | -1.5 | G |  |
|  | rs7115089 | C | G | 0.31 | C | Proxy, rs7941030 |
|  | **rs7134375** | C | A | 0.4 | C |  |
|  | rs3741414 | C | T | 0.46 | C | Proxy, rs11613352 |
|  | rs7134594 | T | C | -0.44 | C |  |
|  | **rs4759375** | C | T | 0.86 | C | Proxy, rs4759377 |
|  | rs4765127 | G | T | 0.44 | G |  |
|  | **rs838880** | T | C | 0.61 | T | NA |
|  | rs1532085 | G | A | 1.45 | G |  |
|  | rs2652834 | G | A | -0.39 | A |  |
|  | rs3764261 | C | A | 3.39 | C |  |
|  | **rs16942887** | G | A | 1.27 | G |  |
|  | rs2925979 | C | T | -0.45 | T |  |
|  | **rs881844** | G | C | -0.51 | C |  |
|  | rs4148008 | C | G | -0.42 | G | NA |
|  | **rs4082919** | T | G | -0.4 | G |  |
|  | rs7241918 | T | G | -1.31 | G |  |
|  | rs12967135 | G | A | -0.42 | A |  |
|  | **rs7255436** | A | C | -0.45 | C |  |
|  | **rs737337** | T | C | -0.64 | C |  |
|  | rs4420638 | A | G | -1.06 | G |  |
|  | **rs386000** | G | C | 0.83 | G | NA |
|  | rs1800961 | C | T | -1.88 | T |  |
|  | rs6065906 | T | C | -0.93 | C | NA |
|  | **rs181362** | C | T | -0.46 | T |  |
|  |  |  |  |  |  |  |
| LDL | **rs12027135** | T | A | -1.1 | T |  |
|  | rs2479409 | A | G | 2.01 | G |  |
|  | rs3850634 | T | G | -1.59 | T | NA |
|  | rs629301 | T | G | -5.65 | T |  |
|  | rs2807834 | G | T | -1.09 | G | NA |
|  | **rs514230** | T | A | -1.13 | T |  |
|  | rs1367117 | G | A | 4.05 | A |  |
|  | rs4299376 | T | G | 2.75 | G |  |
|  | **rs12916** | T | C | 2.45 | C |  |
|  | rs6882076 | C | T | -1.67 | C |  |
|  | **rs3757354** | C | T | -1.43 | C |  |
|  | **rs1800562** | G | A | -2.22 | G |  |
|  | rs3177928 | G | A | 1.83 | A |  |
|  | rs11153594 | C | T | -0.89 | C | Proxy, rs9488822 |
|  | rs1564348 | T | C | 1.95 | C |  |
|  | rs12670798 | T | C | 1.26 | C |  |
|  | **rs217386** | G | A | -1.17 | G | NA |
|  | rs2126259 | C | T | -2.22 | C | Proxy, rs9987289 |
|  | rs1030431 | G | A | 0.95 | A | Proxy, rs2081687 |
|  | rs2954022 | C | A | -1.84 | C | Proxy, rs2954029 |
|  | **rs11136341** | A | G | 1.4 | G | NA |
|  | **rs649129** | C | T | 2.05 | T |  |
|  | rs1129555 | G | A | 1.08 | A | Proxy, rs2255141 |
|  | rs174583 | C | T | -1.71 | C | Proxy, rs174546 |
|  | rs964184 | C | G | 2.85 | G |  |
|  | **rs11220462** | G | A | 1.95 | A |  |
|  | rs11065987 | A | G | -0.97 | A |  |
|  | **rs1169288** | A | C | 1.42 | C |  |
|  | **rs2332328** | C | T | 1.17 | T |  |
|  | rs247616 | C | T | -1.45 | C | Proxy, rs3764261 |
|  | rs2000999 | G | A | 2 | A |  |
|  | **rs7225700** | C | T | -0.87 | C | NA |
|  | **rs6511720** | G | T | -6.99 | G |  |
|  | rs10401969 | T | C | -3.11 | T |  |
|  | rs4420638 | A | G | 7.14 | G |  |
|  | rs2902941 | A | G | -0.98 | A | Proxy, rs2902940 |
|  | rs909802 | C | T | 1.41 | T |  |
|  |  |  |  |  |  |  |
| Total cholesterol | |  |  |  |  |  |
|  | rs12027135 | T | A | -1.22 | T | NA |
|  | rs2479409 | A | G | 1.96 | G | NA |
|  | rs3850634 | T | G | -2.6 | T | NA |
|  | rs7515577 | A | C | -1.18 | A | NA |
|  | rs629301 | T | G | -5.41 | T | NA |
|  | rs2807834 | G | T | -1.38 | G | NA |
|  | rs514230 | T | A | -1.36 | T | NA |
|  | rs1367117 | G | A | 4.16 | A | NA |
|  | rs1260326 | C | T | 1.91 | T | NA |
|  | rs4299376 | T | G | 3.01 | G | NA |
|  | rs6759321 | G | T | 1.18 | T | NA |
|  | rs2290159 | G | C | -1.42 | G | NA |
|  | rs12916 | T | C | 2.84 | C | NA |
|  | rs6882076 | C | T | -1.98 | C | NA |
|  | rs3757354 | C | T | -1.46 | C | NA |
|  | rs1800562 | G | A | -2.16 | G | NA |
|  | rs3177928 | G | A | 2.31 | A | NA |
|  | rs2814982 | C | T | -1.86 | C | NA |
|  | rs9488822 | A | T | -1.18 | A | NA |
|  | rs1564348 | T | C | 2.18 | C | NA |
|  | rs2285942 | C | T | 1.7 | T | NA |
|  | rs2072183 | G | C | 2.01 | C | NA |
|  | rs2126259 | C | T | -3.14 | C | NA |
|  | rs1961456 | A | G | 1.07 | G | NA |
|  | rs1030431 | G | A | 1.26 | A | NA |
|  | rs2737229 | A | C | -1.11 | A | NA |
|  | rs2954022 | C | A | -2.3 | C | NA |
|  | rs11136341 | A | G | 1.34 | G | NA |
|  | rs581080 | C | G | -1.57 | C | NA |
|  | rs1883025 | C | T | -2.24 | C | NA |
|  | rs651007 | C | T | 2.3 | T | NA |
|  | rs2255141 | G | A | 1.14 | A | NA |
|  | rs10832963 | G | T | -1.06 | G | NA |
|  | rs174550 | T | C | -1.78 | T | NA |
|  | rs964184 | C | G | 4.68 | G | NA |
|  | rs7941030 | T | C | 0.97 | C | NA |
|  | rs11220463 | A | T | 2.01 | T | NA |
|  | rs11065987 | A | G | -0.96 | A | NA |
|  | rs1169288 | A | C | 1.45 | C | NA |
|  | rs1532085 | G | A | 1.54 | A | NA |
|  | rs3764261 | C | A | 1.67 | A | NA |
|  | rs2000999 | G | A | 2.34 | A | NA |
|  | rs7206971 | G | A | 1.01 | A | NA |
|  | rs7239867 | G | A | -1.94 | G | NA |
|  | rs6511720 | G | T | -7.09 | G | NA |
|  | rs10401969 | T | C | -4.74 | T | NA |
|  | rs4420638 | A | G | 6.83 | G | NA |
|  | rs492602 | A | G | 1.27 | G | NA |
|  | rs2277862 | C | T | -1.19 | C | NA |
|  | rs2902940 | A | G | -1.38 | A | NA |
|  | rs4297946 | G | C | 1.52 | C | NA |
|  | rs1800961 | C | T | -4.73 | C | NA |
|  |  |  |  |  |  |  |
| Triglycerides | |  |  |  |  |  |
|  | rs2131925 | T | G | -4.94 | T | NA |
|  | rs1321257 | A | G | 2.76 | G | Proxy, rs4846914 |
|  | rs1042034 | T | C | -5.99 | T |  |
|  | rs1260326 | C | T | 8.76 | T |  |
|  | rs10195252 | T | C | -2.01 | T | NA |
|  | rs2943645 | T | C | -1.89 | T | Proxy, rs2972146 |
|  | rs645040 | T | G | -2.22 | T |  |
|  | rs442177 | T | G | -2.25 | T |  |
|  | rs9686661 | C | T | 2.57 | T |  |
|  | rs1553318 | C | G | -2.63 | C | Proxy, rs6882076 |
|  | rs2247056 | C | T | -2.99 | C |  |
|  | rs13238203 | C | T | -7.91 | C | NA |
|  | rs7811265 | A | G | -7.91 | A |  |
|  | rs11776767 | G | C | 2.01 | C |  |
|  | rs1495743 | C | G | 2.97 | G |  |
|  | rs12678919 | A | G | -13.64 | A |  |
|  | rs2954029 | A | T | -5.64 | A |  |
|  | rs10761731 | A | T | -2.38 | A | NA |
|  | rs2068888 | G | A | -2.28 | G |  |
|  | rs174546 | C | T | 3.82 | T |  |
|  | rs964184 | C | G | 16.95 | G |  |
|  | rs11613352 | C | T | -2.7 | C |  |
|  | rs12310367 | A | G | -2.42 | A | Proxy, rs4765127 |
|  | rs2412710 | G | A | 7 | A |  |
|  | rs2929282 | A | T | 5.13 | T |  |
|  | rs261342 | C | G | 2.99 | G | NA |
|  | rs11649653 | C | G | -2.13 | C |  |
|  | rs7205804 | G | A | -2.88 | G | Proxy, rs3764261 |
|  | rs10401969 | T | C | -7.83 | T |  |
|  | rs439401 | C | T | -5.5 | C |  |
|  | rs4810479 | T | C | 3.32 | C |  |
|  | rs5756931 | T | C | -1.54 | T | NA |
|  | - | - | - | - | - | APOE, rs439401 |

Effect sizes as published in Teslovich et al. GWAS[[14](#_ENREF_14)]

HDL and LDL SNPs in bold were used for non-pleiotropic gene scores.[[15](#_ENREF_15)] Differences in the scores used by MDCS are noted.

**S1 References**

1. Harris TB, Launer LJ, Eiriksdottir G, Kjartansson O, Jonsson PV, et al. (2007) Age, Gene/Environment Susceptibility-Reykjavik Study: multidisciplinary applied phenomics. Am J Epidemiol 165: 1076-1087.

2. Alonso A, Agarwal SK, Soliman EZ, Ambrose M, Chamberlain AM, et al. (2009) Incidence of atrial fibrillation in whites and African-Americans: the Atherosclerosis Risk in Communities (ARIC) study. Am Heart J 158: 111-117.

3. Soliman EZ, Prineas RJ, Case LD, Zhang ZM, Goff DC, Jr. (2009) Ethnic distribution of ECG predictors of atrial fibrillation and its impact on understanding the ethnic distribution of ischemic stroke in the Atherosclerosis Risk in Communities (ARIC) study. Stroke 40: 1204-1211.

4. Benjamin EJ, Levy D, Vaziri SM, D'Agostino RB, Belanger AJ, et al. (1994) Independent risk factors for atrial fibrillation in a population-based cohort. The Framingham Heart Study. JAMA 271: 840-844.

5. Kannel WB, Feinleib M, McNamara PM, Garrison RJ, Castelli WP (1979) An investigation of coronary heart disease in families. The Framingham offspring study. Am J Epidemiol 110: 281-290.

6. Smith JG, Platonov PG, Hedblad B, Engstrom G, Melander O (2010) Atrial fibrillation in the Malmo Diet and Cancer study: a study of occurrence, risk factors and diagnostic validity. Eur J Epidemiol 25: 95-102.

7. Heeringa J, van der Kuip DA, Hofman A, Kors JA, van Herpen G, et al. (2006) Prevalence, incidence and lifetime risk of atrial fibrillation: the Rotterdam study. Eur Heart J 27: 949-953.

8. Ridker PM, Chasman DI, Zee RY, Parker A, Rose L, et al. (2008) Rationale, design, and methodology of the Women's Genome Health Study: a genome-wide association study of more than 25,000 initially healthy american women. Clin Chem 54: 249-255.

9. (1989) The Atherosclerosis Risk in Communities (ARIC) Study: design and objectives. The ARIC investigators. Am J Epidemiol 129: 687-702.

10. Kannel WB, Gordon T, Offutt D (1969) Left ventricular hypertrophy by electrocardiogram. Prevalence, incidence, and mortality in the Framingham study. Ann Intern Med 71: 89-105.

11. Feinleib M, Kannel WB, Garrison RJ, McNamara PM, Castelli WP (1975) The Framingham Offspring Study. Design and preliminary data. Prev Med 4: 518-525.

12. Berglund G, Elmstahl S, Janzon L, Larsson SA (1993) The Malmo Diet and Cancer Study. Design and feasibility. J Intern Med 233: 45-51.

13. Hofman A, Breteler MM, van Duijn CM, Janssen HL, Krestin GP, et al. (2009) The Rotterdam Study: 2010 objectives and design update. Eur J Epidemiol 24: 553-572.

14. Teslovich TM, Musunuru K, Smith AV, Edmondson AC, Stylianou IM, et al. (2010) Biological, clinical and population relevance of 95 loci for blood lipids. Nature 466: 707-713.

15. Voight BF, Peloso GM, Orho-Melander M, Frikke-Schmidt R, Barbalic M, et al. (2012) Plasma HDL cholesterol and risk of myocardial infarction: a mendelian randomisation study. Lancet 380: 572-580.
